# A Rare Case of Adult Rhabdomyoma in the Parapharyngeal Space: Diagnostic Challenges and Surgical Management

**DOI:** 10.1155/crip/1626816

**Published:** 2025-10-31

**Authors:** Alkmini Gatsounia, Pinelopi Bosgana, Gerasimos Danielides, Athanasios Vlachodimitropoulos, Hamida Kurabi, Spyridon Lygeros

**Affiliations:** ^1^Department of Otorhinolaryngology-Head and Neck Surgery, General University Hospital of Patras, Patras, Greece; ^2^Department of Pathology, General University Hospital of Patras, Patras, Greece

**Keywords:** adult rhabdomyoma, benign neoplasm, dysphagia, parapharyngeal space, striated muscle tumor, surgical excision

## Abstract

**Introduction:**

Adult rhabdomyoma (ARM) is an exceedingly rare benign neoplasm accounting for less than 2% of all striated muscle tumors. Originating from striated muscle cells, it primarily occurs in the head and neck region but is particularly rare in the parapharyngeal space.

**Case Presentation:**

We report a case of a 64-year-old male patient who presented with a 6-month history of progressive dysphagia and globus pharyngeus. CT and MRI scans revealed a well-defined multilobular mass in the right parapharyngeal space. Surgical excision was performed, and histopathological evaluation confirmed ARM. ARM diagnosis presents a challenge due to nonspecific clinical and radiological manifestations. Histopathological examination remains the gold standard for definitive diagnosis. Surgical excision is the treatment of choice, and close postoperative monitoring is crucial due to high recurrence rates.

**Conclusion:**

This case highlights the diagnostic challenges and treatment modalities for ARM in a rare anatomical location. A multidisciplinary approach incorporating radiological, cytological, and histopathological evaluations is essential for accurate diagnosis and effective management.

## 1. Introduction

Rhabdomyoma is a rare benign neoplasm of mesenchymal lineage, originating from striated muscle cells [1]. These tumors account for less than 2% of all striated muscle neoplasms [2]. Rhabdomyomas are classified based on their anatomical localization, as either cardiac or extracardiac, with the latter being exceedingly rare [3, 4]. Further histological classification identifies three subtypes within extracardiac rhabdomyoma: fetal, genital, and adult [5]. Adult rhabdomyomas (ARMs) and fetal rhabdomyomas predominantly arise from the musculature of the third and fourth branchial arches, explaining their occurrence in the head and neck region—particularly in the parapharyngeal space (PPS) [5, 6]. Their rarity and varied clinical presentation have prompted ongoing study into their pathogenesis, manifestations, and optimal management.

ARM typically presents as a slow-growing, well-circumscribed mass, often associated with symptoms arising from its anatomical location or compressive effects on adjacent structures. In the PPS, these tumors commonly manifest with nonspecific symptoms such as progressive dysphagia and a foreign body sensation. This case highlights the clinical presentation, imaging characteristics, surgical management, and histopathological features of ARM, underscoring that surgical excision remains the treatment of choice despite its benign nature. We describe the successful intraoral excision of a large, multilobular ARM of the PPS, a surgical approach rarely documented for lesions of this size and location, thereby adding valuable insight to existing literature.

## 2. Case Presentation

We hereby report the case of a 64-year-old male patient, with no significant medical history and no history of smoking or alcohol abuse, who was referred to our tertiary specialist unit with a 6-month history of gradually progressive dysphagia. He was also experiencing a painless foreign body sensation in the right side of his throat. Physical examination of the throat revealed bulging of the right tonsil and the right PPS and palpation of the neck revealed a well-defined mass in the right PPS without, however, any palpable lymph node enlargement. At nasoendoscopy, the mobility of the larynx appeared normal.

**Figure 1:**
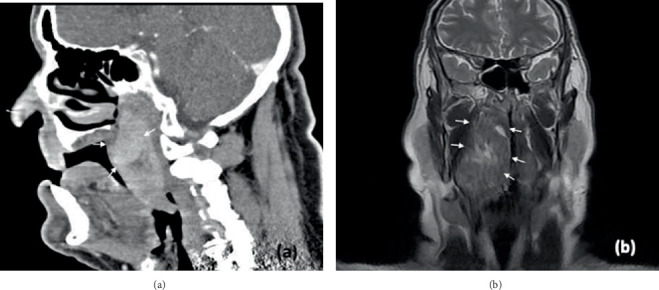
(a) Sagittal view of CT scan demonstrating the parapharyngeal lesion. The image reveals a well-encapsulated, multilobular mass in the right parapharyngeal space. The lesion's boundaries and internal architecture are highlighted, emphasizing its distinction from surrounding tissues. (b) Coronal view of MRI of the parapharyngeal lesion. This flair T2-weighted image delineates the lesion's internal characteristics, including heterogeneity in signal intensity. White arrows are used to highlight the mass.

CT and MRI, after gadolinium contrast substance injection, highlighted a well-defined multilobular mass 6 × 4.5 × 4 cm in the right PPS. On CT, ARM typically appears as a homogeneous, slightly hyperdense lesion (Figure 1a). On MRI, it is isointense or mildly hyperintense relative to skeletal muscle in both T1- and T2-weighted sequences, with homogeneous contrast enhancement (Figure 1b).

**Figure 2:**
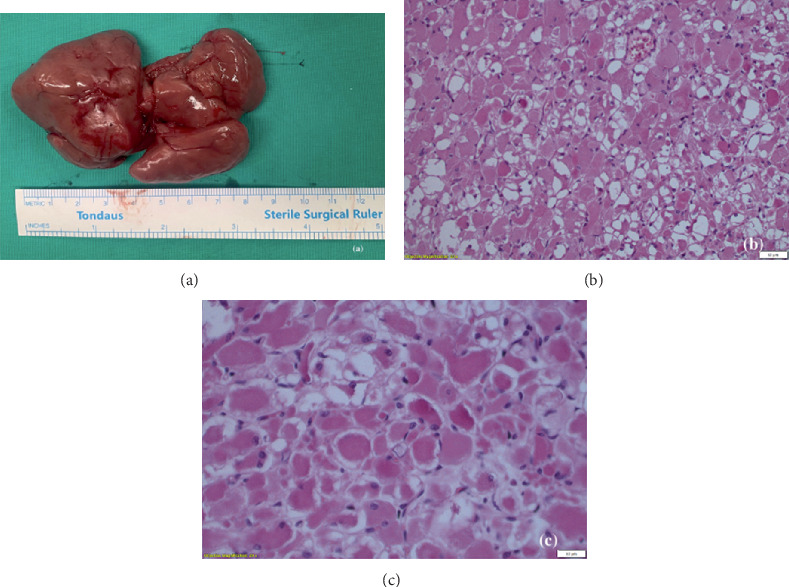
(a) Postexcision image of the multilobular mass that was dissected from the right parapharyngeal space. (b) Tumor composed of cells that resemble skeletal muscle (×200). (c) Round to polygonal cells, with eosinophilic cytoplasm and small nuclei, without atypia or mitoses (×400).

Fine needle aspiration cytology (FNAC) was not performed due to the mass's deep location within the PPS and its proximity to vital structures, including the great cervical vessels, which posed a risk of complications. Given the obstructive symptoms, such as progressive dysphagia and a sensation of a foreign body, surgical excision was chosen as the optimal approach, for both diagnostic (via excision biopsy) and therapeutic purposes. Considering the size and position of the mass relative to the cervical vessels, we performed an intraoral excision alongside a right tonsillectomy under general anesthesia. The excised mass was sent for histopathological examination (Figure 2a).

**Figure 3:**
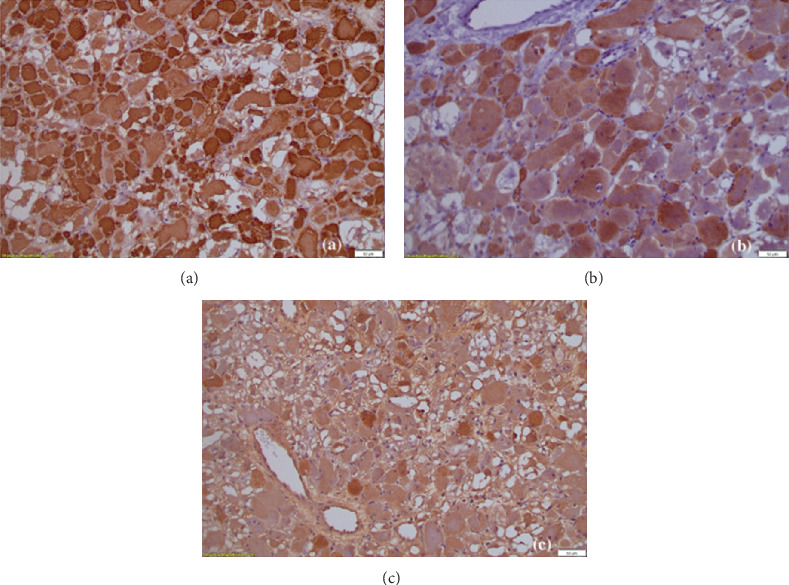
(a) Expression of muscle-specific actin/MSA (×200). (b) Expression of desmin (×200). (c) Expression of myoglobin (×200).

Histological examination revealed a well-circumscribed, not encapsulated, tumor mass composed of sheets of large, well-differentiated, skeletal muscle cells (Figure 2b). The cells were round or polygonal, with abundant eosinophilic cytoplasm and small nuclei, without mitoses or cytological atypia (Figure 2c). On immunohistochemical examination, the cells were positive for MSA, desmin, and myogenin (Figures 3a, 3b, and 3c) and negative for AE1/AE3 and S100. The final diagnosis was ARM, a benign skeletal muscle tumor. The tonsil had signs of chronic inflammation, and the presence of actinomyces was noted. The patient had an uneventful postoperative course, remained in the hospital under antibiotic treatment for 2 days without complications, and was discharged on the third day. He is currently being monitored on an outpatient basis.

## 3. Discussion

Rhabdomyoma is a rare tumor, accounting only for 2% of skeletal muscle tumors [6]. It is commonly found in the head and neck region, with up to 76% of noncardiac cases occurring there [7]. This prevalence may be attributed to the tumor's origin from branchial musculature of the third and fourth branchial arches [8]. In terms of anatomical predilections, ARMs mostly arise in the PPS (35%), followed by the larynx (15%), submandibular region (14%), paratracheal region (12%), tongue (11%), and floor of the mouth (9%) [8]. ARMs show a male predominance (3–5: 1) and usually occur in individuals over 40 years of age [6, 8, 9], with about 20% of cases being multifocal [1, 8].

The PPS is an anatomically complex region of the head and neck where tumors are rare, comprising about 0.5% of all head and neck neoplasms, most of which (82%) are benign [10]. The differential diagnosis for a PPS tumor includes salivary gland neoplasms, neurogenic tumors, and rare tumors such as hemangiomas, adenomas, and lymphomas [11]. Salivary gland neoplasms, mainly pleomorphic adenomas, are most common, followed by neurogenic tumors—primarily schwannomas and paragangliomas—which may be the most frequent benign type in some cases [12]. Other lesions include hemangiomas, branchial cleft cysts, lipomas, and basal cell adenomas [10]. Malignant tumors (< 20%) are typically adenoid cystic carcinomas, squamous cell carcinomas, or lymphomas with rare rhabdomyosarcomas also reported [10, 13]. ARM in the PPS is exceedingly rare, with roughly 150 cases reported [2–4, 13, 14]. It is important to note that the tumors in this region often present with subtle symptoms.

The clinical manifestation of ARM is typically characterized by a gradually enlarging, painless cervical mass, but symptoms vary by location [3]. In the PPS, dysphagia, globus sensation, and hoarseness may occur, though these are nonspecific [2, 15]. Our patient's age and presentation were consistent with prior reports.

Diagnosing rhabdomyoma still remains challenging due to the absence of specific clinical manifestations and imaging, making histopathological examination essential for diagnosis in most cases. Imaging techniques, such as CT and MRI, provide valuable information regarding the tumor's dimensions, location, and benign characteristics [16]. However, radiologic evaluation offers nonspecific data [4, 7]. These imaging modalities typically reveal a well-circumscribed, noninvasive benign mass, but differentiating rhabdomyomas from other entities, such as minor salivary gland neoplasms, can be difficult [1]. Notably, ARMs lack aggressive features like bone invasion or significant necrosis, which are commonly observed in malignant tumors such as sarcomas or aggressive lymphomas [17].

Fine needle aspiration (FNA) can be used for preoperative evaluation, with an 87% sensitivity for detecting malignant disease, and has diagnosed some ARMs solely through cytology [4]. However, it may be inconclusive when sampling surrounding skeletal muscle and other tumors can be mistaken for ARM. In our case, FNA was not performed due to the tumor's deep location near critical structures, deeper than the great cervical vessels, which posed a risk of complications. Benign radiological features on CT and MRI, along with the tumor's size, location, and absence of suspicious lymph nodes, further supported direct surgical excision, serving both diagnostic and therapeutic purposes due to significant obstructive symptoms, including dysphagia and foreign body sensation.

Surgical approaches to PPS tumors include transcervical, transcervical–transparotid, transcervical–transmandibular, and transoral approaches. The transcervical approach is the most common, offering good access to the inferior PPS, while the transcervical–transparotid approach is used for salivary gland neoplasms with parapharyngeal involvement, requiring careful facial nerve preservation [16, 17]. The transcervical–transmandibular approach is reserved for large, recurrent, highly vascular, or skull base–involving tumors, offering superior exposure and vascular control, but with greater morbidity [18].

The transoral approach offers a minimally invasive option, accessing the tumor directly through the oropharynx. It is best suited for small, benign, and avascular tumors confined to the prestyloid region or for biopsy [16]. While this approach provides the benefit of no external scarring, it does have limitations. Specifically, it offers limited control over major vascular structures and carries an increased risk of recurrence due to the potential for incomplete tumor excision [18].

Despite the benign nature of ARM, surgery remains the gold standard, driven by symptom severity, impact on quality of life, and diagnostic needs, with generally safe outcomes and rare complications [3, 19].

The mass in our patient was in the right PPS, confined to the upper portion of the PPS, and demonstrated well-defined benign features on imaging. The absence of vascular involvement or significant extension near the styloid process made the transoral approach a suitable choice, particularly as the surgery also served as an excision biopsy. This approach enabled direct tumor access and excision while minimizing morbidity and avoiding external scarring. While the transoral approach carries inherent risks, such as incomplete excision and recurrence, the tumor's characteristics in this case minimized these concerns. A meticulous surgical technique ensured complete tumor removal, and postoperative follow-up, including clinical examination and nasoendoscopy, showed no signs of residual mass or recurrence, confirming the success of the approach.

Following surgical excision, histopathological examination remains the gold standard for confirming the diagnosis of ARM [20]. Macroscopically, rhabdomyomas are well-defined, lobulated, brown–reddish tumors, with a fibrous consistency, typically demonstrating no invasion of the surrounding tissues [9]. Microscopically, they display specific features that mimic skeletal muscle [20]. The tumor cells exhibit a polygonal shape with small, round nuclei featuring vesicular chromatin. The cytoplasm appears acidophilic and finely granular, accompanied by prominent nucleoli, clearly defined cell borders, and vacuoles containing glycogen [6, 9]. The cytoplasm may also display vacuolated (“spider cells”) or contain rod-like or “jack-stray”–like crystalline structures or cross striations [9]. The neoplastic cells were immunoreactive for desmin, myogenin, and muscle-specific actin, which are markers of differentiated skeletal muscle cells. The presence of muscle-specific proteins, such as myosin and actin, through immunohistochemistry, was pivotal in establishing the diagnosis and distinguishing ARM from other potential differential diagnoses [2, 4].

Although ARM is benign in nature, its high recurrence rate remains a paradox. Postoperative recurrence rates for ARM range between 10% and 42% and are often linked to incomplete surgical excision, particularly in tumors with a multilobulated architecture [2, 6]. In light of this, our patient remains under close surveillance for any signs of local recurrence as long-term monitoring is recommended [9].

## 4. Conclusion

In conclusion, although ARMs are rare, their occurrence in the head and neck region—and particularly within the PPS—requires careful clinical consideration. Optimal management relies on a multidisciplinary approach with imaging and definitive histopathological confirmation, while surgical treatment should be aimed at minimizing morbidity and preserving the patient's quality of life. In this context, the transoral approach represents a valuable option, as demonstrated in our case.

## Data Availability

The data that support the findings of this study are available on request from the corresponding author.

## Patient Perspective

I am very happy with the outcome and grateful to the doctors. Before the surgery, I had great difficulty eating and drinking, and I was very worried about what the tumor might be. Thankfully, it was benign, and I was able to eat normally and quickly return to my daily life after the surgery.
